# Surface-Modified Multifunctional Thymol-Loaded Biodegradable Nanoparticles for Topical Acne Treatment

**DOI:** 10.3390/pharmaceutics13091501

**Published:** 2021-09-18

**Authors:** Camila Folle, Natalia Díaz-Garrido, Elena Sánchez-López, Ana Maria Marqués, Josefa Badia, Laura Baldomà, Marta Espina, Ana Cristina Calpena, María Luisa García

**Affiliations:** 1 Department of Pharmacy and Pharmaceutical Technology and Physical Chemistry, Faculty of Pharmacy and Food Sciences, University of Barcelona, 08028 Barcelona, Spain; cfollefo7@alumnes.ub.edu (C.F.); m.espina@ub.edu (M.E.); anacalpena@ub.edu (A.C.C.); marisagarcia@ub.edu (M.L.G.); 2 Department of Biochemistry and Physiology, Faculty of Pharmacy and Food Sciences, University of Barcelona, 08028 Barcelona, Spain; ndiazgarrido@ub.edu (N.D.-G.); josefabadia@ub.edu (J.B.); lbaldoma@ub.edu (L.B.); 3 Institute of Biomedicine, University of Barcelona, 08028 Barcelona, Spain; 4 Sant Joan de Déu Research Institute (IR-SJD), 08950 Barcelona, Spain; 5 Institute of Nanoscience and Nanotechnology (IN2UB), University of Barcelona, 08028 Barcelona, Spain; 6 Department of Biology, Healthcare and Environment, Faculty of Pharmacy and Food Sciences, University of Barcelona, 08028 Barcelona, Spain; ammarques@ub.edu

**Keywords:** PLGA, thymol, nanoparticles, skin, HaCaT cells, anti-inflammatory, chitosan, phosphatidylcholine, poloxamer

## Abstract

The present work is focused on the development of novel surface-functionalized poly(lactic-co-glycolic acid) nanoparticles loaded with thymol (TH-NPs) for topical administration enhancing thymol anti-inflammatory, antioxidant and wound healing activities against acne. TH-NPs were prepared by solvent evaporation method using different surface functionalization strategies and obtaining suitable physicochemical parameters and a good short-term stability at 4 °C. Moreover, TH-NPs skin penetration and antioxidant activity were assessed in ex vivo pig skin models. Skin penetration of TH-NPs followed the follicular route, independently of the surface charge and they were able to enhance antioxidant capacity. Furthermore, antimicrobial activity against *Cutibacterium acnes* was evaluated in vitro by the suspension test showing improved antibacterial performance. Using human keratinocyte cells (HaCat), cytotoxicity, cellular uptake, antioxidant, anti-inflammatory and wound healing activities were studied. TH-NPs were non-toxic and efficiently internalized inside the cells. In addition, TH-NPs displayed significant anti-inflammatory, antioxidant and wound healing activities, which were highly influenced by TH-NPs surface modifications. Moreover, a synergic activity between TH-NPs and their surface functionalization was demonstrated. To conclude, surface-modified TH-NPs had proven to be suitable to be used as anti-inflammatory, antioxidant and wound healing agents, constituting a promising therapy for treating acne infection and associated inflammation.

## 1. Introduction

Acne vulgaris is one of the most prevalent skin inflammatory disorders affecting 9.4% of the population worldwide [1,2]. It is a multifactorial disease. Their pathophysiology is complex, with both internal and external triggers [3]. This disease is induced by several factors such as irregular keratinocyte proliferation and differentiation, increased sebum production by active sebaceous glands and imbalances in the skin microbiota. This imbalance is particularly related to certain *Cutibacterium* strains, among them *Cutibacterium acnes,* a normal skin commensal previously known as *Propionibacterium acnes* [4]. In addition, exogenous factors such as hormones, drugs, nutrition, stress or smoke habits, can also trigger acne development [5,6]. This combination of factors leads to skin lesions such as whiteheads, blackheads, pustules and cysts developing into swelling and inflammation [7].

*C. acnes* is a normal resident of healthy skin, mainly located surrounding the hair follicle, which is likely to proliferate under unbalanced function of the sebaceous glands, contributing to inflammation and acne development [8]. Hence, *C. acnes* has a dual activity. This skin commensal strain is essential for sebum control and maintenance of the acidic pH of the pilosebaceous follicle by hydrolysing sebum triglycerides and via propionic acid secretion [4,9]. However, it can act as a pathogen under dysbiosis conditions that help its overgrowth in active sebaceous glands [10].

Imbalanced skin microbiome, including *C. acnes* overgrowth, trigger innate immune system activation, leading to cutaneous inflammation. Consequently, the great array of induced immune-regulatory and pro-inflammatory mediators amplify direct damaging effects on molecules and cells, including DNA, proteins and lipids, causing immunosuppression [11].

Skin inflammation is an innate and non-specific skin immunological response towards external and internal environment modifications or aggressions. The skin immunological activity includes microbial ligands for toll-like receptors (TLR). Host responses to gram-positive bacteria peptidoglycan and gram-negative bacterial lipopolysaccharide are mediated by TLR-2 and TLR-4, respectively [12]. The skin anti-inflammatory mechanism of action consists in modulating the expression of certain genes in order to produce an increase in anti-inflammatory proteins and inhibit pro-inflammatory cytokines release. Cytokines are small proteins which modulate immune responses, regulate cell activation and proliferation. They are produced by epithelial cells, macrophages, CD4 and CD8 T cells. On keratinocytes, *C. acnes* activates TLR-2 and TLR-4 leading to the activation of MAPK and NF-kB pathways. Moreover, they produce interleukins such as (IL)-1, IL-6, IL-8 and TNF-α. Then, ROS production is stimulated, when CD-36 recognizes *C. acnes*, clearing away the bacteria and inducing the inflammation [13]. In acute inflammation, these mediators are present for short periods of time, whereas in chronic inflammation, there is an imbalanced production. Chemokines are cytokine subtypes that take on monocytes/macrophages, neutrophils and T cells from the circulation to the areas of infection. Moreover, neutrophils promote wound healing due to secretion of cytokines, chemokines and growth factors to abolish bacteria and adjust wound microenvironment through oxygen metabolism [14].

Furthermore, sebum composition is severely altered in acne, the ROS produced by neutrophils are involved in the irritation and disruption of the follicular wall, leading to a progressive inflammatory response. ROS overflow might be associated to the lipids present in the sebum. A mediated production of monounsaturated oleic acid is a crucial step in virulence factor of biofilm formation, playing a critical role for bacterial adherence, since it has been found to be an essential nutrient for resident *C. acnes* microbiota [15]. It is also known to enhance epidermal calcium influx in keratinocytes, inducing abnormal keratinization and barrier function associated with increased release of IL-1α in comedones [16]. Furthermore, uric acid increases IL-1β expression through a TLR4-mediated pathway. Thus, increased sebum in acne may activate uric acid–mediated inflammasome. Hyper-keratinization might be initiated via IL-1, suggesting keratinocyte activation cycles, and hence, hyper-proliferation [17]. Hyper-keratinization leads to sebaceous gland obstruction clogging the follicle in function of lipids, bacteria and induced cytokines. In addition to *C. acnes* being a gram-positive bacterium, it possesses a featured cell wall and outer envelope that synthesizes phosphatidylinositol. Its surrounding peptidoglycan containing a cross-linkage peptide chain may allow recognition of receptors contributing to inflammation, secreting TNF-α, IL-1α and IL-8 [12]. In particular, IL-8 together with other factors, may attract neutrophils to the pilosebaceous unit. 

Superoxide or hydroxyl ions, and hydrogen peroxide are highly reactive molecules generated in normal cell metabolism. Moreover, acne-related strains generate ROS and raise inflammation in keratinocytes. Then, it can cause oxidative damage to proteins, lipids, enzymes and DNA [18,19]. These factors reflect on impaired skin function, thus an inflammatory response and cell death. Therefore, the skin is constantly exposed to induced oxidative stress. However, intrinsic antioxidant defence mechanisms contribute to barrier integrity, which is essential for a healthy skin. The cellular redox environment plays a key role in skin homeostasis, preventing oxidative damage of lipids and proteins, and avoiding an imbalanced pro-oxidant/antioxidant stimulus. The oxidation of phenolic compounds normally takes place in the cytosol, in contact with peroxidase/H_2_O_2_. This would produce phenoxyl radical and may co-oxidize glutathione [20]. Consecutive inflammation may affect the skin internal components and their functions. Moreover, cicatrisation processes rely on cell regeneration while healing the infection and the inflammatory responses. 

Natural anti-inflammatory, antioxidants and antimicrobial agents could be the key for preventing or ameliorating acne associated symptoms [21]. In this area, natural compounds are gaining increased importance. Among them, Thymol (TH) is a monoterpene with a phenolic structure associated with several activities such as antioxidant, antimicrobial, antifungal, antiseptic as well as anti-inflammatory [22,23,24]. Despite its multifunctional properties, TH present a low penetration trough skin which could decrease its potential effects against acne. Moreover, TH commercial use is still underexplored, probably because its low water solubility, high volatility and high light sensibility and its low solubility in water [25,26].

In order to overcome TH physicochemical drawbacks, several authors have attempted to encapsulate it into several delivery platforms such as cyclodextrins [26,27], and lipid nanoparticles [25] among others [28]. Moreover, in order to improve active compounds bioavailability after topical administration, polymeric nanoparticles (NPs) constitute excellent potential candidates. Specially for acne treatment due to their small particle diameter, able to penetrate the skin inside the follicle. Additionally, NPs enable the encapsulated active compounds for long-term release inside the lesions. Among the most widely used polymers, poly(lactic-co-glycolic acid) (PLGA) has been approved by the Food and Drug Administration and is one of the most successful biodegradable polymers [29]. Despite their great potential, to our knowledge, to date no other group have developed TH loaded PLGA nanoparticles. In addition, PLGA negative surface is highly versatile and can be modified using several compounds in order to improve NPs performance after topical skin delivery. In this area, Chitosan is a natural polysaccharide, positively charged, that has known anti-inflammatory and wound healing activities [30]. Chitosan can be used either as polymer carrier or be adhered on the surface of other types of negatively charged NPs [31]. In addition, some types of natural phospholipids such as phosphatidylcholine that are normally used to produce liposomes, also have demonstrated to possess antioxidant or anti-inflammatory activities enhancing the efficacy of some encapsulated drugs [32]. Moreover, some synthetic types of surfactants such as Poloxamers, have antioxidant and anti-inflammatory activities [33,34].

Therefore, this study was developed to designed thymol-loaded polymeric NPs (TH-NPs) assessing several surface functionalization compounds and comparing their activity in vitro and ex vivo for the treatment of acne. TH-NPs were functionalized either with phosphatidylcholine, Poloxamer 188 or Poloxamer 407. Additionally, combination of chitosan and Poloxamer surface functionalization was also carried out. Bacterial infection inflammation was induced by *C. acnes* inoculation in keratinocyte cells (HaCaT). Moreover, the antioxidant properties, cell regeneration and wound healing activities were also assayed comparing the different formulations developed.

## 2. Materials and Methods

### 2.1. Materials

PLGA Resomer^®^ RG 504H (consisting of a carboxylic terminal group, molecular weight 38,000–54,000 Da and molar ratio lactide:glycolide 50:50) was purchased from Boehringer Ingelheim (Ingelheim am Rhein, Germany). Thymol (TH), Poloxamer 188 (P) and Poloxamer 407 (PP) were purchased from Sigma Aldrich (Madrid, Spain). Chitosan (C) was supplied by HMC+ (GmbH, Saale, Germany), and phosphatidylcholine (L) was acquired from Lipoid^®^ (GmbH, Ludwigshafen am Rhein, Germany). Double distilled water was used after filtration in a Millipore system. All other chemicals and reagents used in the study were of analytical grade.

### 2.2. Methods

#### 2.2.1. Preparation of Thymol Loaded Nanoparticles

Thymol-loaded PLGA NPs (TH-NPs) containing a matrix structure (nanospheres) were obtained by solvent displacement evaporation, as described by Fessi et al. [35]. In the current study, a previously optimized formulation based on TH-PLGA-NPs was modified by functionalizing TH-NPs surface using several compounds. In order to prepare the NPs, the organic phase composed by PLGA and 2.5 mg/mL of TH was dissolved in acetone and the aqueous phase consisted on either phosphatidylcholine (TH-NP-L-) or Poloxamer 188 (TH-NP-P-) or Poloxamer 407 (TH-NP-PP-), for negatively charged particles. Additionally, positively charged particles were also produced containing chitosan (TH-NP-P-C+, TH-NP-PP-C+), where the aqueous solution contained 1% acetic acid. The organic phase was added dropwise into the aqueous phase, under continuous stirring. A rotatory evaporator (Buchi, Flawil, Switzerland) under constant pressure was used to evaporate the organic phase, obtaining the nanoparticles. Empty NPs (B-NPs) were prepared using the same procedure but without the addition of TH.

#### 2.2.2. Nanoparticles Physicochemical Characterization

The average particle size (Z_av_) and polydispersity index (PI) were determined by photon correlation spectroscopy, using a ZetaSizer Nano ZS (Malvern Instruments; Malvern, UK). The surface charge, measured as zeta-potential (ZP), was determined by electrophoretic mobility using the same instrument.

Encapsulation of TH was measured indirectly by quantification of unloaded amount. Samples were diluted 1:10 in Milli-Q water:ethanol (90:10) and centrifuged (Centrifuge 5415C, Geratebau Eppendorf GmbH, Engelsdorf, Germany) for 10 min at 14,000 rpm, using Millipore filter device (Amicon^®^ Ultra, 0.5 mL 100 K, Merck Millipore Ltd., Carrigtwohill Co. Cork IRL, Darmstadt, Germany). The filtered fractions were quantified by HPLC, and the EE was determined by the Equation (1):

EE = (Ci − Cs)/Ci·100 (1)

where Ci is the initial concentration of the active and Cs is the concentration of the unloaded amount found in the filtered fraction. 

HPLC quantitative analysis was performed by reverse-phase high-performance liquid chromatography (HPLC) by a modification of the method described previously [36]. Studies were carried out in Acquity Waters System with UV detector, using a Kromasil^®^ column (C18, 5 μm, 150 mm × 4.6 mm), (Teknokroma, Barcelona, Spain). The mobile phase consisted of acetonitrile:water under gradient conditions of 30:70/58:42/30:70 during 20 min. TH was determined at wavelength of 275 nm.

#### 2.2.3. Stability of Thymol Loaded Nanoparticles

Short-term stability of TH NPs was evaluated after one month of storage at 4, 25, 30 and 40 °C by measuring Z_av_, PI, ZP and pH. Additional long-term studies were carried out during 6 months by measuring the physicochemical parameters monthly at 4 °C. Moreover, backscattering profiles of TH-NPs stored at 4 °C, were also recorded using TurbiscanLab^®^ (Formulation, Toulousse, France) [37].

#### 2.2.4. Ex Vivo Skin Penetration Route of Thymol Loaded Nanoparticles

Ex vivo pig skin was obtained from the animal house (Bellvitge, University of Barcelona), used in accordance with the protocol approved by the Ethics Committee of the University of Barcelona.

Prior to the experiment, rhodamine-labelled PLGA (R-PLGA) was synthesized as described by Gonzalez-Pizarro et al. [38] and used at 0.01% obtaining rhodamine labelled nanoparticles (R-TH-NPs). Skin penetration assay was executed using vertical diffusion Franz cells (FDC-400, Vidra-Foc, Barcelona, Spain) with a thermal bath set to 32 °C to mimic skin in vivo conditions, under constant stirring. R-TH-NPs were applied onto ex vivo pig skin 0.64 cm^2^ (donor compartment) and penetration was allowed for 24 h. Skin samples were washed, fixed in PBS containing 4% paraformaldehyde (PFA) for 4 h, followed by cryoprotection into PBS with 30% sucrose for 24 h, snap-frozen in isopentane at −50 °C and then kept overnight at −80 °C. Samples were mounted in O.C.T. ^®^ Compound (Tissue-Tek^®^, Sakura Finetek, Torrance, CA, USA) and sliced with cryostat microtome (LEICA CM3050 S) at −20 °C onto glass-slides Superfrost^®^ Plus (Menzel-Glaser, Thermo Scientific, USA), covered with Fluoromount G^®^ (Invitrogen, Thermo Fisher Scientific, Rockford, IL, USA). Samples were visualized by confocal laser scanning microscopy (Zeiss LSM 880). Images were acquired using Zen Black 2.3 software performing z-stack sections and thus processed with ImageJ software v1.53k.

#### 2.2.5. Ex Vivo Skin Antioxidant Activity by Methylene Blue Reduction

A colorimetric assay was performed using methylene blue dye to test the ex vivo antioxidant activity of TH and surface functionalized TH-NPs. Methylene blue, in combination with an antioxidant molecule, reduces into a colorless lecomethylene blue [39].

Pig skin samples were cut into 2 cm^2^ and placed into a 6-well plate containing 0.5 mL of PBS, with the stratum corneum (SC) facing up. Then, a methylene blue solution at 0.01% was applied on the surface of each skin sample and incubated for 4 h at 32 °C, in the presence of humidity. Skin fragments were washed with PBS and the SC was dried with filter paper. TH-NPs were applied onto the skin (30 µL) and further incubated for 1 h. The control sample was treated with distilled water. Images were recorded at initial and 1 h post-treatment to assess differences in methylene blue reduction.

#### 2.2.6. Free-Radical Scavenging by DPPH

The scavenging capacity of TH, surface functionalized TH-NPs and surface functionalization components was evaluated using DPPH (2,2-diphenyl-1-picrylhydrazyl) assay, based on other authors with some modifications [40]. Samples were dissolved and diluted in methanol at concentrations ranging from 0.1 to 10 mg/mL, and DPPH, a free radical compound, was prepared in 80% methanol at 0.1 mM. Sample dilutions were transferred into a 96-well plate (200 µL/well) and 20 µL of the DPPH stock solution was added into each well. BHT (butyl-hydroxytoluene), a known antioxidant compound was used as endogenous control.

Samples without DPPH were used as blank. Samples were incubated in the dark for 45 min on a mechanical shaker. The UV-VIS absorbance was measured at 517 nm and data were calculated using the Equation (2):

(2) $$Free\;Radical\;Scavening\left(\%\right)=\frac{Ac-\left(As-Ab\right)}{Ac}\cdot 100$$

where *Ac, As* and *Ab are* the absorbances of the control, sample and blank, respectively.

#### 2.2.7. Antimicrobial Efficacy of Thymol Loaded Nanoparticles

A fresh inoculum of *C. acnes* was prepared in PBS adjusted to an optical density of 0.72 at 550 nm, using a UV-visible spectrophotometer (Shimadzu Corp., Kyoto, Japan). The assay was carried out in a total volume of 1 mL containing TH or TH-NPs (900 µL) at a final concentration of 250 µg/mL and fresh bacterial inoculum (100 µL). Samples were kept at 37 °C in a shaker incubator (Innova 4080, New Brunswick Scientific, Edison, NJ, USA) for 30 min. Then, 100 µL of each test tube was neutralized in 900 µL of Berens cosmetic diluent (Scharlab, Barcelona, Spain) for 15 min [41]. Ten-fold dilutions in PBS (10 µL) were added to clostridium reinforced medium agar dishes for enumerating bacteria by the drop count method. Microbial count was performed after incubation for 48 h under anaerobic conditions at 37 °C. Bacterial viability was expressed as CFU/mL against time (h). 

#### 2.2.8. Cytotoxicity and Cellular Uptake of Thymol Loaded Nanoparticles

Human keratinocytes (HaCaT) cells were cultured in high glucose Dulbecco’s Modified Eagle’s Medium (DMEM) from Thermofisher, supplemented with 10% fetal bovine serum (FBS), 2 mM L-glutamine, 100 units/mL penicillin G and 100 µg/mL streptomycin. Cells were incubated at 37 °C and 5% CO_2_ and experiments were performed when cells reached 80–90% of confluence.

Cytotoxicity of TH-NPs was evaluated by the MTT (3-(4,5-Dimethylthiazol-2-yl)-2,5-diphenyl tetrazolium bromide) assay that is based on the mitochondrial reduction of tetrazolium salt by intracellular dehydrogenases of viable cells. Samples were tested at concentrations up to 2, 10 and 20 µg/mL. Briefly, HaCaT cells were seeded in 96-well plates (100 μL) at a density of 2 × 10^5^ cells/well, adjusted in an automated cell counter (Countess, Invitrogen, Thermofisher) and grown for 24 h at 37 °C. Then, TH-NPs were added at the indicated concentrations and cells were further incubated for 24 h. Finally, the medium was removed and MTT (Sigma-Aldrich Chemical Co, St. Louis, MO, USA) was added at 0.25% in PBS. After 2 h incubation, the medium was replaced by 100 µL DMSO (99% dimethyl sulfoxide, Sigma-Aldrich, Madrid, Spain) [42]. Cell viability was then measured at 570 nm in a Modulus^®^ Microplate Photometer (Turner BioSystems Inc., Sunnyvale, CA, USA). Results were expressed as percentage of cell survival relative to untreated cells.

HaCaT cells were seeded in an 8-well µ-slide (Ibidi^®^). Cells were incubated in FBS/phenol red-free medium in the presence or absence of TH-NPs for 2 h at the indicated concentration. Cell membrane was stained with wheat germ agglutinin (WGA) Alexa-488 (Molecular Probes) at 1 µg/mL for 15 min followed by fixation with 3% paraformaldehyde for 25 min. Cell nuclei were stained with 4′,6-diamidino-2-phenylindole (DAPI, Sigma Aldrich, Madrid, Spain) at 0.5 µg/mL for 15 min. Internalization of NPs in HaCaT cells was assessed by confocal microscopy (Leica TCS SPII), using the 63X oil immersion objective lens [38]. Images were processed using Fiji image software [38].

#### 2.2.9. Anti-Inflammatory Activity in TNF-α-Induced Inflammation Model 

HaCaT cells were seeded in 12-well plates at a density of 2 × 10^5^ cells/well and grown until 80–90% confluence. Cells were then treated with TH-NPs for 2 h, followed by stimulation with 50 µM TNF-α for 2 h to induce inflammation [43]. The medium was replaced by fresh FSB-free medium and cells were incubated overnight. Supernatants were collected, and quantification of secreted interleukin-6 (IL-6) was carried out by using ELISA Human kit (BD OptEIA^®^ Set Human IL-6, BD Biosciences, Franklin Lakes, NJ, USA) following manufacturer instructions. Absorbance was measured at 450 and 560 nm using a plate reader (Varioskan, Thermo Fisher Scientific, Rockford, IL, USA). Data were processed and analyzed using version 5 GraphPad^®^ Prism software.

#### 2.2.10. Anti-Inflammatory Activity in *C. acnes*-Induced Inflammation Model 

This experiment was performed as described in the previous section, but *C. acnes* fresh inoculum was added instead of TNF-α. *C. acnes* was grown until the stationary phase (5 days incubation under anaerobiosis in BHI culture medium). Then bacterial cells were harvested and diluted in FSB-free DMEM medium, adjusted to OD 1.2 at 550 nm. Different dilutions of this inoculum were added to HaCaT cells and incubated overnight. Quantification of IL-6 in cell culture supernatants was assayed by using ELISA Human kit (BD OptEIA^®^ Set Human IL-6, BD Biosciences, Franklin Lakes, NJ, USA) following manufacturer instructions [44].

#### 2.2.11. Real-Time Quantitative Polymerase Chain Reaction (RT-qPCR)

HaCaT cells were adjusted to a density of 2 × 10^5^ cells/well and seeded in 12-well plates. After 48 h, cells were treated with TH or TH-NPs or blank NPs (B-NPs) for 2 h. Next, cells were stimulated for 4 h with *C. acnes* prepared in FBS-free DMEM medium, adjusted to OD 1.2 at 550 nm. HaCaT cells without any treatment were used as a negative control and cells incubated only with *C. acnes* as a positive control. Total RNA was isolated from cells using an RNA extraction kit (Qiagen RNeasy, Germantown, MD, USA) following the manufacturer’s guide (Qiagen, Crawley, UK) and quantified by the ratio of absorbance values at 260 and 280 nm using a NanoDrop TM-2000 spectrophotometer (Thermo Fisher Scientific, Waltham, MA, USA). cDNA was synthesized from RNA (1 µg) by using the High-Capacity cDNA Reverse Transcription kit (Applied Biosystems, Foster City, CA, USA) in a final volume of 20 µL. Quantitative PCR reactions were performed in a StepOne Plus PCR cycler (Applied Biosystems, Foster City, CA, USA) by using SYBR^®^ Green PCR Master Mix (Applied Biosystems, Foster City, CA, USA) and specific human oligonucleotide primers for IL-1α, IL-1β, IL-6, IL-8, TNF-α and β-actin (endogenous control, primers specified in Table S1 of Supplementary Materials). Control reactions were performed in the absence of RNA. The standard PCR program was conducted by one denaturation cycle for 10 min at 95 °C followed by 40 cycles of 15 s at 95 °C and 1 min at 60 °C. Relative gene expression was calculated as fold change compared to sample control by means of 2−ΔΔCt formula [42].

#### 2.2.12. Antioxidant Activity Assessed by ROS Quantification 

HaCaT cells were seeded in 96-well plates at 2 × 10^5^ cells/well (100 µL) for 72 h. Cells were loaded with the fluorogenic dye H_2_DCFDA (2′,7′-dichlorodihydrofluorescein diacetate) at 25 µM diluted in phenol red/FBS-free DMEM medium, for 45 min in the dark. This fluorogenic dye passively diffuses into the cells, is deacetylated by intracellular esterase and emits fluorescence upon oxidation by reactive oxygen species (ROS) [45]. Then, cells were washed with PBS and incubated for 2 h with TH, surface-functionalization compounds, functionalized TH-NPs or B-NPs. At this time, 10 µL of 20 mM H_2_O_2_ were added to the wells. Fluorescence was measured at excitation and emission wavelengths of 485 and 530 nm, respectively. Data were acquired at 0, 30, 60 and 120 min. The data of the positive control (H_2_O_2_) after 2 h was used to normalize values (%). Background fluorescence of control cells were subtracted from all measurements.

#### 2.2.13. Wound Healing Activity in HaCaT Cells by the Scratch Assay

In order to study wound healing activity of the developed TH-NPs, prevention and treatment were assessed. In order to study wound healing prevention, HaCaT cells were seeded in 12-well plates at a density of 5 × 10^4^ cells/well and grown for 24 h until 70–80% confluence. Cells were treated for 2 h with free TH, surface-functionalized TH-NPs or functionalization compounds, washed with PBS and further incubated for 24 h. Then, scratches were performed in the middle of each well using a 200 µL pipette tip, washed with PBS, refilled with FBS-free culture medium and incubated for 24 h [46,47]. Contrast phase images of the scratches were obtained at the beginning of the experiment (T0) and after 24 h using a fluorescent microscope, and the wound area was measured using ImageJ software.

In the previous assay, the capacity to prevent wound healing was assessed, whereas in a second assay, wound healing treatment was examined by applying the formulations after the lesion was caused. For this second experiment, HaCaT cells were seeded and grown for 24 h. After creating the scratches as previously mentioned, cells were washed with PBS and refilled with DMEM containing 1% FBS. Images at this timepoint were recorded by using fluorescent microscope at 10X (LEICA DFC300FX). Cells were then treated with either TH, surface functionalized TH-NP or surface functionalization compounds for 2 h and further incubated for 24 h in 1% FBS—culture medium [30]. Images at 24 h were recorded and processed using ImageJ software.

## 3. Results and Discussion

### 3.1. Thymol Loaded Nanoparticles Physicochemical Characterization

TH-NPs were prepared using the solvent displacement method and surface functionalized with different compounds (TH-NP-L-, TH-NP-P-; TH-NP-PP-, TH-NP-P-C+, TH-NP-PP-C+). Their physical-chemistry characterization is shown in Table 1. All formulations presented good homogeneity below 0.2 indicating monodisperse systems and high entrapment efficacy values around 80% leading to 2 mg/mL of TH encapsulated inside the NP [48]. Moreover, Z_av_ was adequate for skin topical administration and pH was slightly acidic in all the cases. Regarding ZP, TH-NP-L-, TH-NP-P-; TH-NP-PP- show negative surface charges but the formulations functionalized with chitosan showed highly positive surface charge which may be able to favor interaction with negatively charged skin tissues.

### 3.2. Stability of Thymol Loaded Nanoparticles

**Figure 1:**
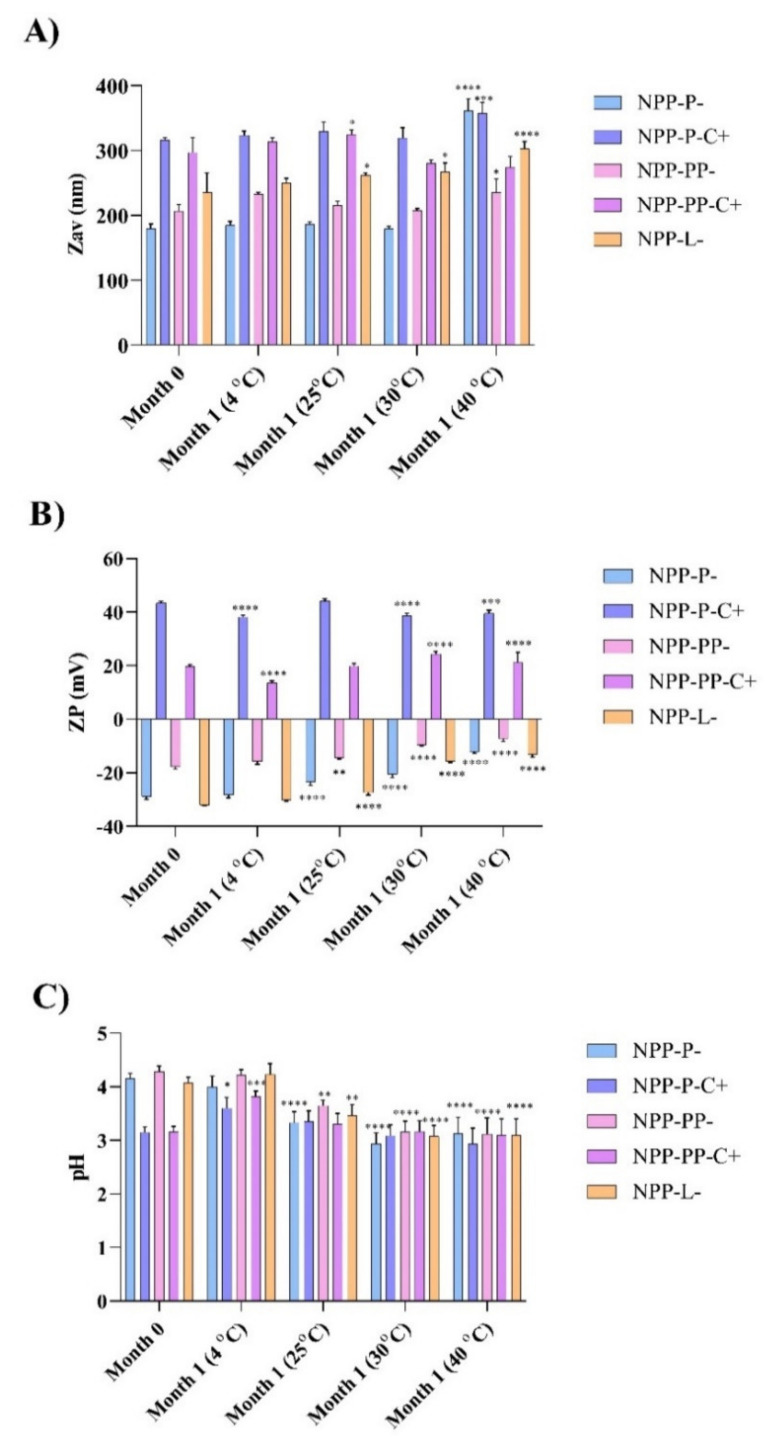
One-month stability of TH NPs with different surface functionalization strategies at 4, 25, 30 and 40 °C. (**A**) Average size, (**B**) Zeta potential (ZP), (**C**) pH values. Statistical significance was analyzed against freshly prepared formulations (one month), * *p* < 0.5; ** *p* < 0.01; *** *p* < 0.001; **** *p* < 0.0001.

TH-NPs stability with different surface functionalization compounds (TH-NP-L-, TH-NP-P-; TH-NP-PP-, TH-NP-P-C+, TH-NP-PP-C+) were measured in order to assess their short-term stability. Physical-chemical properties were measured after one-month of storage at 4, 25, 30 and 40 °C. As can be observed in Figure 1, after 1 month it was corroborated that the most suitable storage temperature was 4 °C since no statistically significant differences were observed regarding the average size of TH-NPs. Even though ZP and pH of chitosan functionalized TH-NPs show significant differences after one month, its values are still adequate for topical administration. In addition, it can be observed that at higher temperatures such as 30 or 40 °C, all the parameters vary significantly as it has been previously reported by other authors developing PLGA NPs [49,50].

Since the most suitable temperature for surface functionalized TH-NPs storage was found to be 4 °C, 6-month stability was studied at this temperature. Results obtained in Table 2 show differences in the stability of the different functionalized NPs. It can be observed that after 6 months, NPP-P- vary its parameters. Even though NPP-P- Z_av_ remains below 200 nm and PI below 0.1, ZP decreases significantly after 6 months of storage. A similar behavior is observed when chitosan was added to the formulation (NPP-P-C+) observing stability until 3-months of storage. NPP-PP- also show a ZP decrease after 3 months although their chitosan functionalized formulations (NPP-PP-C+) not vary their properties until 6 months. In the case of NPP-L-, NPs modified their physicochemical properties prior to 3 months after their preparation. 

**Figure 2:**
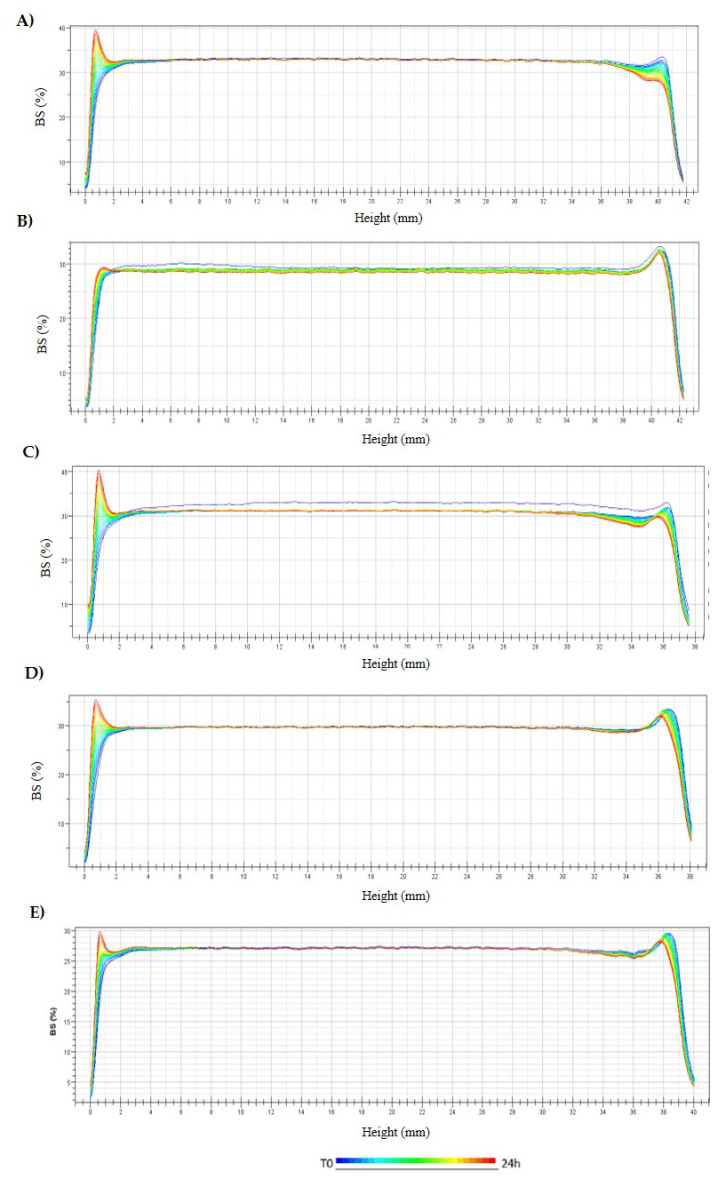
Backscattering profile of NPs measured monthly for 6 months after storage at 4 °C. (**A**) TH-NP-L-, (**B**) TH-NP-P-, (**C**) TH-NP-P-C+, (**D**) TH-NP-PP- and (**E**) TH-NP-PP-C+.

In addition, backscattering profile of surface-functionalized TH-NPs was analyzed by Turbiscan^®^ and results are shown in Figure 2. All optimized TH-NPs underwent a slight sedimentation that was reversible by agitation. This sedimentation might be the cause of the physicochemical modifications previously observed (Table 2). Moreover, since the difference between the obtained profiles was below 10%, this indicates a suitable stability of all the functionalized TH-NPs. However, in order to ensure long-term stability, TH-NPs lyophilization and incorporation into semi-solid formulations would be contemplated in further studies.

### 3.3. Ex Vivo Skin Penetration of Thymol Loaded Nanoparticles

**Figure 3:**
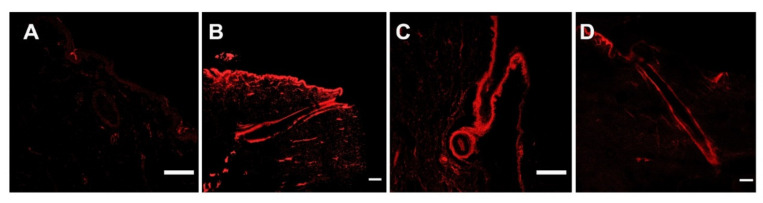
Pig skin hair follicle penetration of R-TH-NPs in 24 h by confocal microscopy. (**A**) untreated (control), (**B**) R-TH-NP-L-, (**C**) R-TH-NP-P- and (**D**) R-TH-NP-P+. Scale bar: 200 µm.

As can be observed in Figure 3, R-TH-NPs successfully penetrated into the skin hair follicle within 24 h. This is of extreme relevance since it is the main site where acne associated infection and inflammation occurs. Moreover, it can be observed that penetration was not influenced by the surface charge carried out since both negatively and positively charge TH NPs were able to penetrate through the skin follicle ex vivo.

### 3.4. Ex Vivo Methylene Blue Reduction

**Figure 4:**
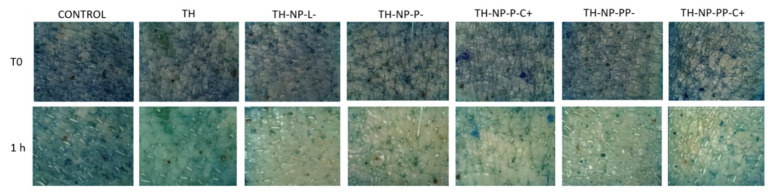
Ex vivo antioxidant activity by methylene blue reduction in pig skin. Images recorded at time 0 and 1 h of the studied compounds (control, free TH, TH-NP-L-, TH-NP-P-, TH-NP-P-C+, TH-NP-PP- and TH-NP-PP-C+).

The antioxidant efficiency of TH and surface functionalized TH-NPs was evaluated in the ex vivo pig skin model by measuring methylene blue reduction, which results in a colorless compound (Figure 4). Results showed that all the assessed TH-NPs showed antioxidant activity, greater than that of free TH. No qualitative differences between TH-NPs were observed.

### 3.5. In Vitro Antioxidant Activity

**Figure 5:**
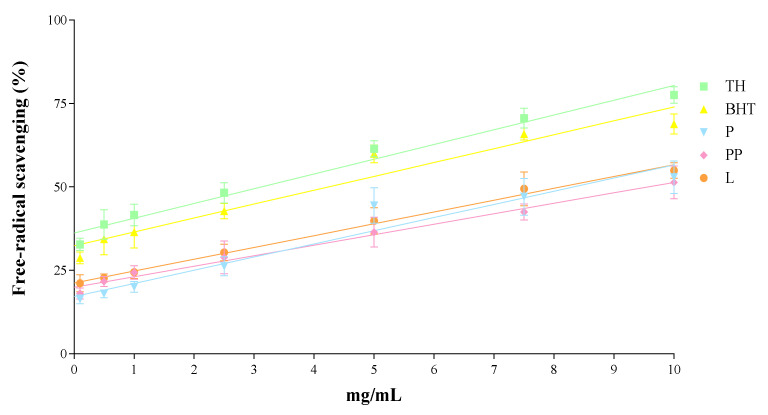
Antioxidant activity of TH, BHT and surface compounds alone (P, PP and L) assessed by the DPPH free-radical scavenging assay. The 100% ROS was obtained by the value of the control (H_2_O_2_) in 2 h.

The in vitro antioxidant activity of the different compounds used to prepare the formulations was evaluated individually by the DPPH assay (Figure 5). Results expressed as free radical scavenging capacity (%) showed that TH has similar antioxidant activity as the control BHT, although slightly higher. When tested separately, the surface compounds P, PP and L displayed slightly in vitro free radical scavenging activity, although it was lower than the activity of TH and BHT in all dosages tested. 

### 3.6. In Vitro Antimicrobial Efficacy

**Figure 6:**
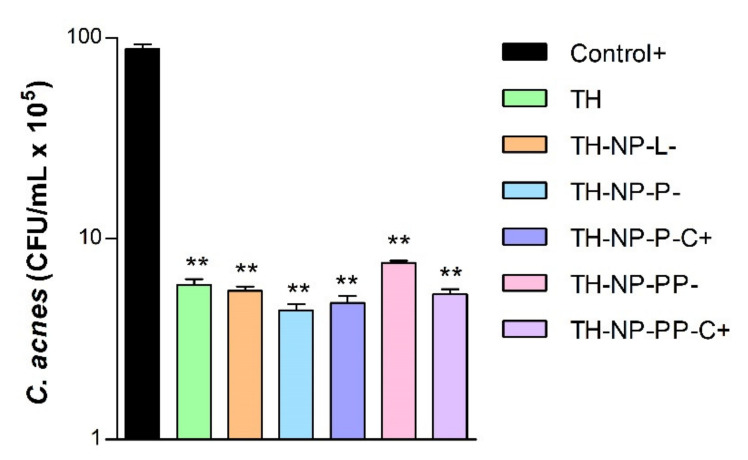
Antimicrobial activity of NPs against *C. acnes* measured by the suspension test. Values represent microbial counts in CFU/mL after 30 min incubation and are expressed as Mean ± SD (*n* = 3). Statistical analysis was carried out via one-way ANOVA, with Tukey’s Multiple Comparison Test: **, *p* < 0.001 against control (*C. acnes* without any treatment).

The in vitro antimicrobial activity of optimized surface functionalized TH-NPs against *C. acnes* was similar to that of TH and in all the cases, statistical signifcant differences were obtained against the positive control (Figure 6). The higher antimicrobial activity was obtained with TH-NP-P-, although no statistically significant differences were observed between the different formulations.

### 3.7. Cytotoxicity and Cellular Uptake of Thymol Loaded NPs in HaCaT Cells

Cytotoxicity of TH-NPs was evaluated on HaCaT cells using the MTT assay. Cells were incubated for 24 h with TH or each TH-NP at concentrations of 2, 10 or 20 µg/mL. The surface compounds alone (P, PP, L) were tested at concentrations equivalent to those present in each formulation. Results showed that none of the samples were cytotoxic as cell viability was kept close to the untreated control cells, above 90% (data not shown).

**Figure 7:**
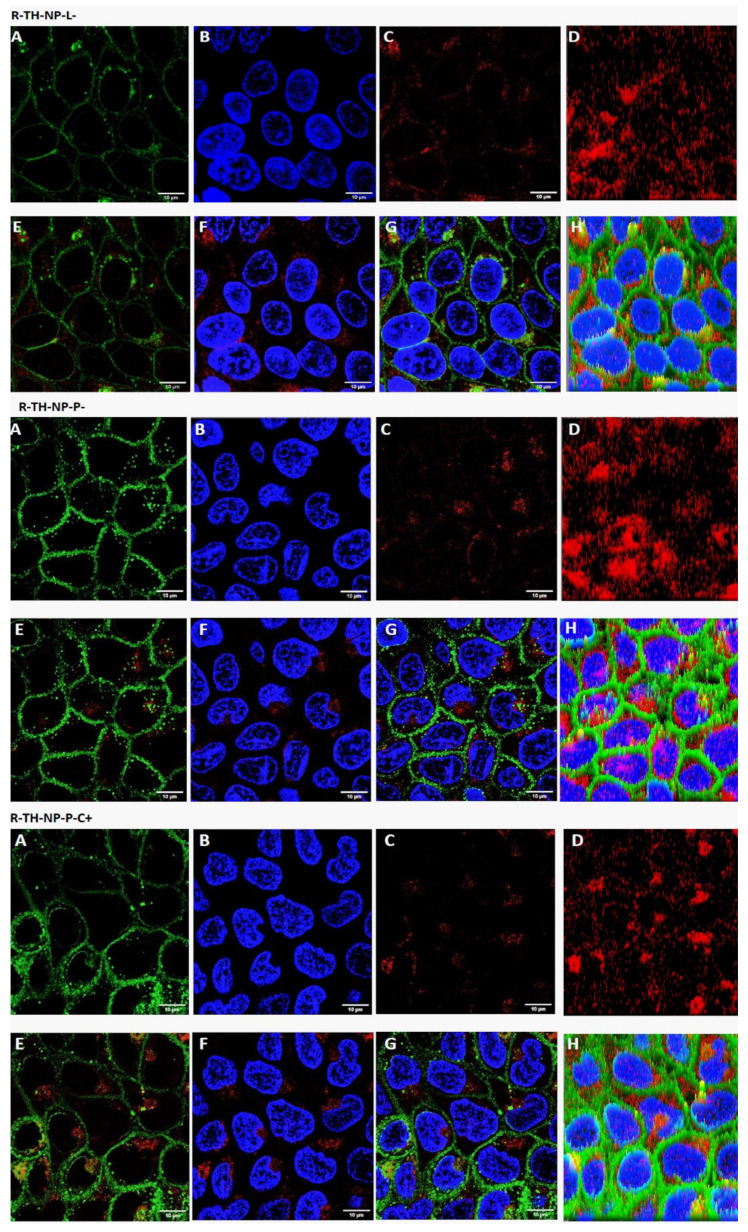
Cellular uptake by confocal microscopy analysis of HaCaT cells incubated with the indicated rhodamine-labelled NPs. (**A**) membrane staining with WGA, (**B**) nuclei staining with DAPI; (**C**) fluorescence of internalized R-TH-NPs, (**D**) 3D-plot of C, (**E**) merged A and C, (**F**) merged B and C, (**G**) merged A, B and C, (**H**) 3D-plot of G. Figure scale bar corresponds to 10 μm.

Cellular uptake was evaluated for R-TH-NP-L-, R-TH-NP-P- and R-TH-NP-P-C+ (20 µg/mL) in HaCaT cells in order to evaluate composition and surface charge influence on cellular uptake. After 2 h incubation, TH-NPs-associated fluorescence was detected by confocal microscopy in cells treated with any of the R-TH-NPs tested (Figure 7). The cell membrane was stained with WGA (green) and the nucleus (blue) with DAPI. In the merged images the internalized TH-NPs were mainly localized in the cytoplasm in all the cases.

### 3.8. Anti-Inflammatory Activity of Thymol Loaded NPs in HaCaT Cells Treated with TNF-α 

**Figure 8:**
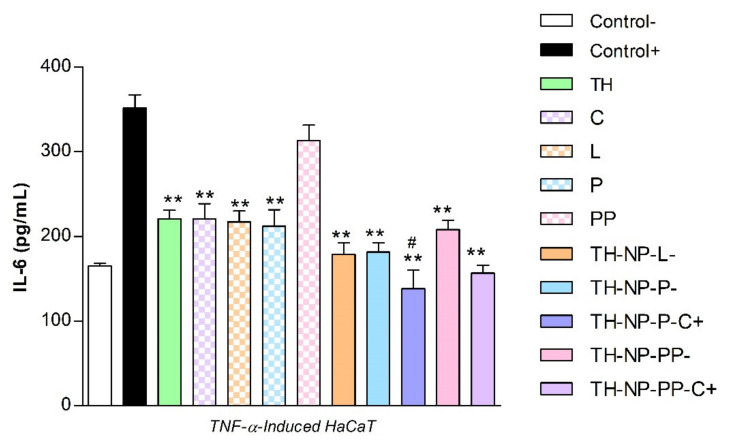
Quantification of secreted IL-6 by ELISA in TNF-α-stimulated HaCaT cells pre-incubated with formulated NPs and free compounds. Values of IL-6 (pg/mL) are the Mean ± SD, *n* = 3. Negative control: HaCaT cells without any treatment; Positive control: HaCaT cells treated only with TNF-α. Statistical analysis was performed by via one-way ANOVA, followed by Tukey’s Multiple Comparison Test. ** *p* < 0.001 compared to positive control, and ^#^ *p* < 0.01 compared to TH.

The anti-inflammatory activity of the formulated NPs was evaluated in the TNF-α-induced inflammation model using HaCaT cells. Secretion of IL-6 was quantified by ELISA in cell supernatants of untreated control HaCaT cells (basal IL-6 expression), TNFα-treated HaCaT cells in the absence (positive control of inflammation) or in the presence of the different NPs. Free TH and surface compounds (C, L, P, PP) were also tested in parallel (Figure 8). Results showed that TH significantly reduced TNF-α-induced secretion of IL-6. All surface compounds analyzed individually, except PP, have similar anti-inflammatory activity as TH. Additionally, all TH-NPs, except TH-NP-PP-, presented higher anti-inflammatory activity than free compounds. The most effective NPs were the positively charged formulations, containing C. However, only TH-NP-P-C+ displayed a significant difference compared to TH.

### 3.9. Anti-Inflammatory Activity of Thymol Loaded NPs in HaCaT Cells Treated with C. acnes 

The inflammatory activity of *C. acnes* was assessed in HaCaT cells treated with different dilutions of a *C. acnes* stock inoculum prepared in DMEM medium (OD 1.2 at 550 nm). Inflammation was evaluated by quantification of secreted IL-8 by ELISA. Results showed that *C. acnes* triggered IL-8 secretion in a dose- dependent manner (Figure S1 of Supplementary Materials). 

**Figure 9:**
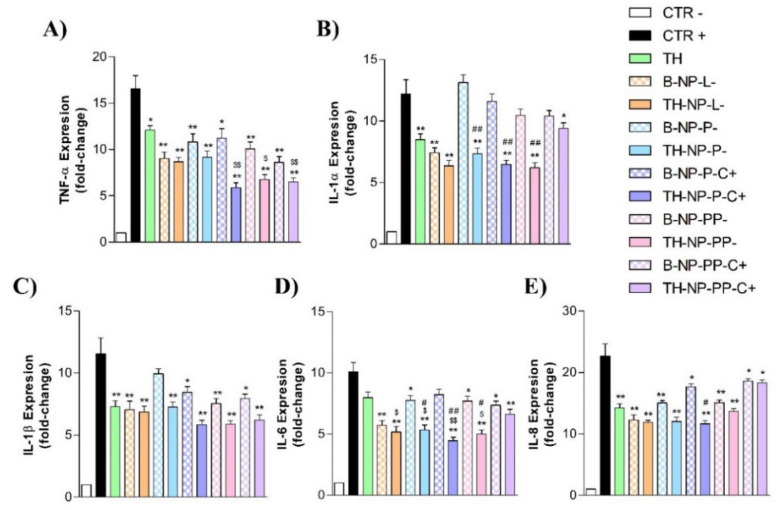
Gene expression levels of inflammatory cytokines in *C. acnes*-infected HaCaT cells. Before the addition of undiluted *C. acnes* inoculum (adjusted to OD 1.2 at 550 nm), HaCaT cells were pre-incubated with TH or the indicated NPs. Relative mRNA levels of (**A**) IL-6, (**B**) IL-8, (**C**) IL-1α, (**D**) IL-1β and (**E**) TNF-α were measured by RT-qPCR, using β-actin as the reference gene. Values (Mean ± SEM, *n* = 3) are expressed as fold-change compared to untreated HaCaT cells (control-). Statistical analysis was performed via one-way ANOVA, followed by Tukey’s Multiple Comparison Test (*p* < 0.05* or *p* < 0.001**): versus positive control (control+); (*p* < 0.05^$^ or *p* < 0.001^$$^): versus TH and (*p* < 0.05^#^ or *p* < 0.001^##^): versus the respective B-NP.

From these results, the undiluted *C. acnes* stock inoculum prepared in DMEM medium (OD 1.2 at 550 nm) was optimized to be added directly to HaCaT cells to induce inflammation in further experiments aimed at evaluating the anti-inflammatory potential of TH-NPs. In this context, expression of genes encoding the inflammatory cytokines TNF-α, IL-1α, IL-1β, IL-6 and IL-8 was analyzed by RT-qPCR after *C. acnes* infection in HaCaT cells pre-treated with TH, TH-NPs or B-NPs (NPs without TH). Cells challenged only with *C. acnes* were used as a positive control of the inflammatory response. Results are illustrated in Figure 9. Infection by *C. acnes* strongly induced the expression of all pro-inflammatory cytokines tested. According to its anti-inflammatory properties, TH significantly decreased the expression of all of them.

Infection by *C. acnes* strongly induced the expression of all pro-inflammatory cytokines tested (positive control). According to its anti-inflammatory properties, TH significantly decreased the expression of all of them. In general, all TH-NPs have anti-inflammatory activity, being able to reduce the mRNA levels of the different cytokines to a greater or lesser degree depending on the type of TH-NPs and the cytokine analyzed. Some TH-NPs have an anti-inflammatory activity greater than that of free TH. Statistically significant differences with respect the reduction caused by TH were apparent when analyzing the expression of IL-1α and IL-6. In the case of the TH-NP-P-C + formulation, statistical differences with respect to TH were also significant for TNF-α and IL-8. The results showed that B-NPS can also reduce significantly the expression of all cytokines except IL-1α. However, the anti-inflammatory activity was less than that exhibited by the equivalent TH-NP. The only TH-NP that did not show significant differences with respect the equivalent B-NP is the TH-NP-L formulation.

### 3.10. Antioxidant Activity via ROS Quantification in H_2_O_2_-Induce H2DCFDALabelled HaCaT

**Figure 10:**
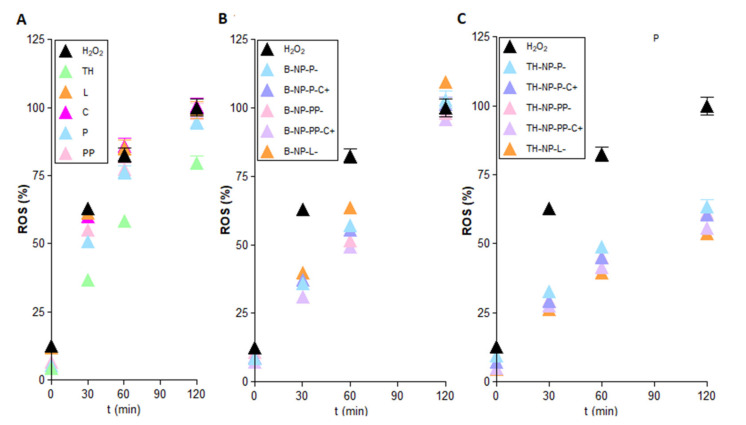
Antioxidant activity of (**A**) TH and free surface compounds, (**B**) B-NPs (blank NPs) and (**C**) TH-NPs evaluated in HaCaT cells challenged with H_2_O_2_. ROS were quantified using the fluorescent probe H2DCFDA. Data are expressed as the Mean ± SD (*n* = 8) of the amount of quantified ROS (%), assigning the value of 100 to the amount of ROS generated after 120 min treatment with H_2_O_2_.

The antioxidant activity of TH-NPs was evaluated by ROS quantification in HaCaT cells stressed with hydrogen peroxide. Treatment with any of the surface funtionalized TH-NPs significantly reduced intracellular ROS to a greater extent than TH (Figure 10). TH displayed antioxidant activity at all assay times (Figure 10A). Moreover, the surface compounds tested individually did not show significant antioxidant activity (Figure 10A), indicating their inability to act as free radical scavengers at the cellular level. In the case of the B-NPs (Figure 10B), they showed a ROS scavenging activity similar to TH up to 60 min after H_2_O_2_ challenge. Even though no significant differences were found, TH-NP-L- showed an increased antioxidant activity that may be due to phosphaditilcholine coating potentiation of TH antioxidant effects [51].

However, this activity was lost at 120 min of incubation since ROS values were similar to that of H_2_O_2_-treated cells. Therefore, surface functionalization compounds could increase TH-NPs antioxidant activity at the cellular level potentiating their effects. In the other hand, all TH-NPs displayed greater antioxidant activity than TH and B-NPs, with minimal variances between them (Figure 10C).

### 3.11. Wound Healing Activity

**Figure 11:**
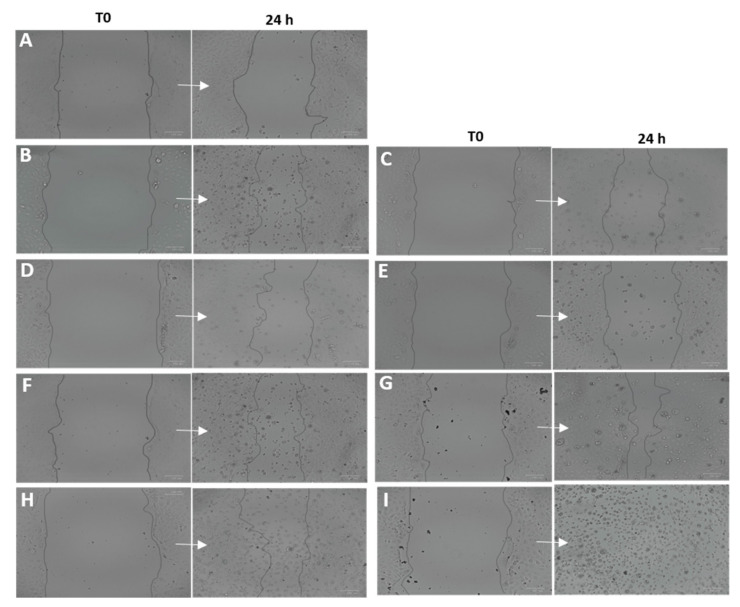
Wound healing activity in HaCaT pre-scratch treatment (wound healing prevention). Scratches were monitored at T0 and after 24 h incubation. (**A**) control, (**B**) Free TH, (**C**) chitosan, (**D**) phosphatidylcholine, (**E**) TH-NP-L-, (**F**) TH-NP-P-, (**G**) TH-NP-P-C+, (**H**) TH-NP-PP- and (**I**) TH-NP-PP-C+.

In this study, the wound healing activity of the samples was analyzed in HaCaT cells following two different protocols. The first study was focused on studying prevention wound healing capacity. In this experiment, a 2 h-treatment with TH-NPs and the coating compounds was applied 24 h prior to the scratch (Figure 11). Images were recorded at the time of the scratch (T0) and after 24 h incubation. Results showed that all samples increased cell regeneration and possess wound healing activity compared to control. TH-NPs (Figure 11E–I) presented higher activity than TH (Figure 11B), specially un the case of TH-NP-PP-C+. The surface compounds alone had minor effects. Therefore, using this assay, wound healing prevention of surface modified TH-NPs has been demonstrated.

**Figure 12:**
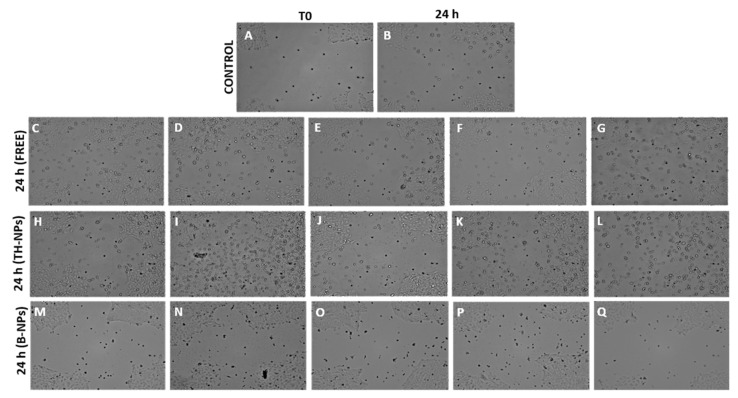
Recorded images of wound healing activity in HaCaT cells under post-scratch treatment (wound healing treatment). (**A**) Control T0, (**B**) Control after 24 h incubation, (**C**) Free TH, (**D**) Poloxamer 188, (**E**) poloxamer 407, (**F**) chitosan, (**G**) phosphatidylcholine, (**H**) TH-NP-P-, (**I**) TH-NP-P-C+, (**J**) TH-NP-PP-, (**K**) TH-NP-PP-C+, (**L**) TH-NP-L-, (**M**) B-TH-NP-P-, (**N**) B-NP-P-C+, (**O**) B-NP-PP-, (**P**) B-NP-PP-C+, (**Q**) TH-NP-L-.

The second protocol was performed in order to study wound healing treatment. Figure 12 illustrates cell regeneration and wound healing efficacy after 24 h incubation. Results indicate that surface-functionalized TH-NPs showed greater wound healing activity than control TH and all other individually tested compounds. Additionally, depending on their surface composition, an increase in cell proliferation and wound closure could be observed specially in the case of Chitosan coated TH-NPs. Therefore, this test confirms that TH-NPs could also be used for wound healing treatment. Meanwhile, B-NPs and surface compounds alone presented minimal activity thus indicating synergic effect between TH and surface functionalization compounds.

## 4. Discussion

In the present study, surface-functionalized PLGA NPs were successfully loaded with thymol by the solvent displacement method in order to avoid TH instability and high volatility. To compare their pharmacological activities against acne, 5 formulations were developed, namely TH-NP-P-, TH-NP-P-C+, TH-NP-PP-, TH-NP-PP-C+ and TH-NP-L-. All the resulting TH-NPs presented suitable physical chemical parameters and a suitable short-term stability at 4 °C. The sedimentation phenomena observed by the backscattering signal was reversed by agitation. Confocal microscopy qualitative analysis confirmed that TH-NPs penetrated into the hair follicle, independent of their surface-coating type or charge. Some authors stated that the reservoir of the hair follicle could store actives 10 times longer than the reservoir of the SC and that hair follicle under movement (in vivo) should improve NPs penetration [52]. Other authors have already described that NPs accumulate in the follicular entry, which is confirmed in the case of surface-functionalized TH-NPs [53,54,55]. Regarding TH-NP-L, it appears that they can penetrate not only in the follicle, by also within the primary layers of the epidermis. This can be explained by the phospholipidic nature of the surface that diffuses easier through the skin. In the case of R-TH-NP-P-, their penetration was found mainly through in the entire hair follicle and a lower amount remaining in the SC. This indicated that this type of surface is favored by the follicular and not the intercellular pathway. On the other hand, concerning R-TH-NP-P-C+, which are positively charged and present slightly higher particle diameter, the penetration could be observed only inside the follicle. This is in accordance with the fact that NPs penetration into hair follicle is size and surface dependent. 

Furthermore, the free-radical scavenging of TH was similar to endogenous control BHT and much higher than the free surface compounds themselves, which showed lower activity. The antimicrobial activity of TH and TH-NPs against *C. acnes* demonstrated to reduce microbial viability within only 30 min incubation. All tested samples presented similar activity, where the highest was achieved for TH-NP-P-. Moreover, since the penetration route of TH-NPs into the skin is through the hair follicle, the observed activity will presumably be performed directly on the acne lesion. 

In HaCaT cell line, TH-NPs and all free compounds themselves did not alter cell viability, presenting no cytotoxicity. The cellular uptake images showed most of the NPs within only 2 h in the cytosol but also present the nucleus, especially for TH-NP-P-. The anti-inflammatory activity of TH and TH-NPs all presented significant reduction on gene expressions tested, where, in all the cases, all or most of TH-NPs performed significant reduction compared to the control and to their corresponding B-NP. Depending on the gene analyzed, the activity was enhanced for a different surface composition of the NPs. Therefore, they act as good booster of the activity. The antioxidant activity in HaCaT cells was achieved for all TH-NPs, higher than TH and all other tested samples. The antioxidant activity of compounds enables cell proliferation faster, that can enhance wound healing processes to improve skin healing process on acne lesions [56]. For the wound healing activity, all scratched treated cells provided higher cell proliferation than control cells. Interestingly, in both prevention and treatment of wound healing activity all surface modified TH-NPs showed suitable results. All TH-NPs provided good cell regeneration, meanwhile the B-NPs showed minimal activity. The antioxidant properties of surface compounds might have influenced on the healing activity, possibly by synergic activity with TH. All TH-NPs showed better results than TH, confirming that surface modified TH-NPs present higher healing capacity than free antioxidant compounds. The antioxidant activity was also confirmed by the methylene blue reduction in ex vivo pig skin, showing that TH-NPs had greater activity than TH within 1 h incubation. 

To summarize, TH was successfully encapsulated into PLGA NPs with suitable particle diameters and good stability. Moreover, other authors have developed different types of NPs for TH encapsulation such as PLA or chitosan NPs obtaining lower stability values (around 60%) [28]. Lipid NPs containing thymol have also been developed with a negative surface charge confirming their anti-inflammatory properties but they were assessed for psoriasis treatment [25]. In addition, Pires and colleagues studied compatibility of TH with several excipients confirming that P80 was a suitable excipient [57]. Moreover, surface-functionalized TH-NPs successfully penetrated into the skin through the hair follicle, where acne occurs. Even though the antimicrobial activity of TH-NPs was similar to TH, they provided outstanding activities as anti-inflammatory, antioxidant and wound healing, when compared to than TH and B-NPs. TH-NPs in contact with HaCaT cells showed that they were able to penetrate inside the cells, enabling greater activities than when compounds were introduced as its free form. TH and TH-NPs presented good anti-inflammatory activity by significant reduction on the gene expression tested. The efficacy of TH-NPs varied depending on their surface coating as well as genes analyzed. The antioxidant activity was proven in HaCaT and on ex vivo skin, and in all cases, TH-NPs were greater than TH. For this reason, they have also provided excellent wound closure and cell proliferation results, where the NPs positively charged, performed higher synergic activity on healing processes. Therefore, our study demonstrates that TH-NPs with surface functionalization using different approaches could constitute a potential efficient treatment for acne. However, these results would need to be assessed in in vivo experiments due to the multifactorial triggers of acne as well as the sebaceous content that can influence NPs stability [58].

## 5. Conclusions

Surface-modified TH-NPs demonstrated to possess antimicrobial, anti-inflammatory, antioxidant and wound healing properties. In addition to TH-NPs multifunctional therapeutic benefits, they penetrate through the hair follicle and, therefore, they could be suitable for the treatment of severe acne disease.

## Figures and Tables

**Table 1:** Surface functionalized formulations of Thymol loaded PLGA NPs (TH-NP-L-: TH-NPs functionalized with phosphatidylcholine; TH-NP-P-: TH-NPs functionalized with Poloxamer 188; TH-NP-PP-: TH-NPs functionalized with Poloxamer 407; TH-NP-P-C+: TH-NPs functionalized with Poloxamer 188 and Chitosan; TH-NP-PP-C+: TH-NPs functionalized with Poloxamer 407 and Chitosan).

	Z_av_ ± SD (nm)	PI ± SD	ZP ± SD (mV)	pH ± SD
NPP-P-	180.2 ± 6.5	0.066 ± 0.037	−28.9 ± 1.0	4.15 ± 0.10
NPP-P-C+	316.5 ± 3.8	0.123 ± 0.035	43.4 ± 0.6	3.15 ± 0.10
NPP-PP-	206.9 ± 9.9	0.101 ± 0.041	−17.8 ± 0.7	4.29 ± 0.10
NPP-PP-C+	297.3 ± 22.9	0.150 ± 0.068	19.6 ± 0.8	3.16 ± 0.10
NPP-L-	235.8 ± 29.7	0.063 ± 0.018	−32.1 ± 0.2	4.08 ± 0.10

**Table 2:** Physicochemical values of TH-NPs with different surface functionalization stored at 4 °C.

	Month	Z_av_ (nm) ± SD	PI ± SD	ZP ± SD (mV)
NPP-P-	0	172.9 ± 1.9	0.066 ± 0.037	−24.5 ± 0.9
	1	177.7 ± 2.2	0.071 ± 0.015	−20.9 ± 0.5
	3	183.8 ± 3.6	0.082 ± 0.005	−18.3 ± 0.7
	6	191.5 ± 1.5	0.091 ± 0.015	−14.3 ± 0.6
NPP-P-C+	0	337.3 ± 7.4	0.123 ± 0.035	23.6 ± 0.3
	1	365.4 ± 3.2	0.141 ± 0.011	23.2 ± 0.9
	3	392.9 ± 8.0	0.158 ± 0.037	21.0 ± 0.3
	6	419.6 ± 11.5	0.197 ± 0.066	17.2 ± 0.2
NPP-PP-	0	184.0 ± 0.9	0.101 ± 0.041	−22.2 ± 0.6
	1	191.2 ± 0.7	0.098 ± 0.022	−18.2 ± 0.6
	3	189.1 ± 10.1	0.099 ± 0.015	−12.1 ± 0.4
	6	220.0 ± 10.8	0.123 ± 0.33	−8.4 ± 0.7
NPP-PP-C+	0	221.1 ± 3.3	0.149 ± 0.036	10.5 ± 0.6
	1	224.1 ± 5.3	0.150 ± 0.068	9.8 ± 0.6
	3	256.1 ± 5.3	0.152 ± 0.077	7.3 ± 0.7
	6	348.5 ± 17.2	0.189 ± 0.023	6.1 ± 0.5
NPP-L-	0	178.5 ± 0.6	0.063 ± 0.018	−41.2 ± 1.8
	1	180.9 ± 1.4	0.076 ± 0.019	−39.7 ± 0.5
	3	214.7 ± 2.7	0.114 ± 0.032	−29.7 ± 0.5
	6	201.9 ± 3.4	0.132 ± 0.027	−23.9 ± 0.8

## Supplementary Materials

The following are available online at https://www.mdpi.com/article/10.3390/pharmaceutics13091501/s1, Table S1: Oligonucleotinde primers used for RT-qPCR, Figure S1: Dose-dependent inflammatory capacity of *C. acnes*. HaCaT cells were incubated for 24 with the indicated dilutions of the *C. acnes* stock inoculum (adjusted to OD 1.2 at 550 nm). Value 1 indicates no dilution. Secreted IL-8 was quantified in the cell culture supernatant by ELISA. Values of IL-8 (pg/mL) are the Mean ± SD, n = 3.
