# Supplementary material for: Surface-Modified Multifunctional Thymol-Loaded Biodegradable Nanoparticles for Topical Acne Treatment
Source: Pharmaceutics. 2021 Sep 18;13(9):1501. doi: 10.3390/pharmaceutics13091501 (PMC8471012; doi:10.3390/pharmaceutics13091501)
Supplement: Supplementary file 1 [file pharmaceutics-13-01501-s001.zip › pharmaceutics-1362172-supplementary.pdf]

pharmaceutics

Supplementary Materials: Surface-Modified Multifunctional Thymol-Loaded Biodegradable Nanoparticles for Topical Acne Treatment

Camila Folle, Natalia Díaz-Garrido, Elena Sánchez-López, Ana Maria Marqués, Josefa Badia, Laura Baldomà, Marta Espina, Ana Cristina Calpena and María Luisa García

Table S1. Oligonucleotinde primers used for RT-qPCR.

Gene	Forward (5′-3′)	Reverse (5′-3′)
TNF-α	CTGCTGCACTTTGGAGTGAT	AGATGATCTGACTGCCTGGG
IL-1α	GAAGAGACGGTTGAGTTTAAGCC	CAGGAAGCTAAAAGGTGCTGA
IL-1β	GTGGCAATGAGGATGACTTGTTTC	TAGTGGTGTCGGAGATTCGTA
IL-8	CTGATTTCTGCAGCTCTGTG	GGGTGGAAAGGTTTGGAGTATG
IL-6	AGCCACTCACCTCTTCAGAAC	GCCTCTTTGCTGCTTTCACAC
Keratin 16 (K16)	CAGCGAACTGGTACAGAGCA	GTTCTCCAGGGATGCTTTCA

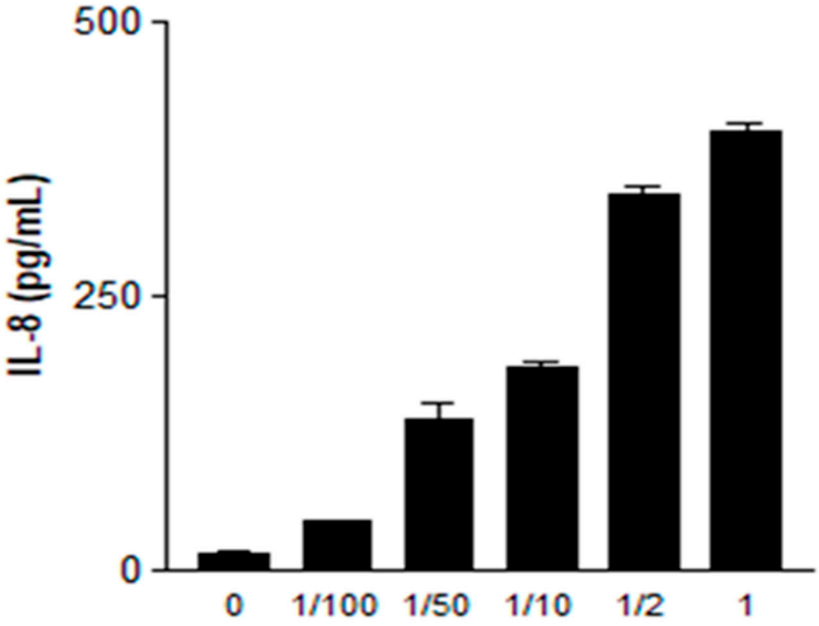

Figure S1. Dose-dependent inflammatory capacity of *C. acnes*. HaCaT cells were incubated for 24 with the indicated dilutions of the *C. acnes* stock inoculum (adjusted to OD 1.2 at 550 nm). Value 1 indicates no dilution. Secreted IL-8 was quantified in the cell culture supernatant by ELISA. Values of IL-8 (pg/mL) are the Mean ± SD, n=3.
